# Wayfinding of Firefighters in Dark and Complex Environments

**DOI:** 10.3390/ijerph18158014

**Published:** 2021-07-29

**Authors:** Beckham Shih-Ming Lin, Ching-Yuan Lin, Chun-Wei Kung, Yong-Jun Lin, Chung-Chyi Chou, Ying-Ji Chuang, Gary Li-Kai Hsiao

**Affiliations:** 1 Department of Architecture, National Taiwan University of Science and Technology, Taipei City 106335, Taiwan; linmin5040@gmail.com (B.S.-M.L.); linyuan@mail.ntust.edu.tw (C.-Y.L.); d9413005@gmail.com (Y.-J.C.); 2 Department of Environmental Engineering, Da-Yeh University, Changhua 515006, Taiwan; gghttr700@gmail.com; 3 Center for Weather Climate and Disaster Research, National Taiwan University, Taipei City 10617, Taiwan; vovman@gmail.com; 4 Department of Fire and Safety, Da-Yeh University, Changhua 515006, Taiwan; dustin@mail.dyu.edu.tw; 5 Department of Disaster Management, Taiwan Police College, Taipei City 11696, Taiwan

**Keywords:** firefighter, dark environment, confined space, wayfinding, complex environment

## Abstract

Firefighters searching in dark and complex environments might lose their orientation and endanger themselves at the fireground. This study conducted experiments in the Training Facility of the New Taipei City Fire Department (NTFD), Taiwan. The objective of the experiments was to analyze the profile of each firefighter by a 13-factor self-report survey and their wayfinding time in dark and complex environments (DCEs). The results showed that age might be a marginally significant factor, and fear of confinement might be a significant factor that could affect firefighters’ wayfinding time in the DCEs. The findings could provide strategies for improving the safety of firefighters working in such environments.

## 1. Introduction

The present study conducted experiments in the Dark, Confined, and Complex Search Training Facility of the New Taipei City Fire Department (NTFD) in Taiwan. The objective was to analyze the profiles of firefighters through a self-report survey and to determine which factors might influence their performance in wayfinding in dark and complex environments (DCEs).

When on a mission, both professional and volunteer firefighters are exposed to environments that require physical and mental balance and special protective equipment [1,2]. Before firefighters arrive at a fire scene, the behavior of trapped civilians is critical to their survival rate [3,4,5,6].

“Heroes” is the image of firefighters in the eyes of civilians. According to an annual statistical report from the Taiwan National Fire Agency, fire departments in Taiwan receive more than 1.11 million calls per year. From 1997 to 2020, a total of 84 firefighter deaths in the country were classified as deaths in the line of duty [7]. Although the number of fires in Taiwan has been decreasing annually, the death rate of firefighters has shown an increasing trend. On 28 April 2018, a fire at a printed circuit board factory in Taoyuan City resulted in the death of six firefighters [8]. In 2015, a fire incident at the Hsin Wu Bowling Alley in Taoyuan City also led to the deaths of six firefighters [9]. In June 2016, a warehouse fire in Hong Kong burned for 108 h, causing injury to 12 firefighters and resulting in them being admitted to the hospital; two of them were declared dead in the emergency room.

A previous study analyzed US firefighters’ search and rescue strategies including the path, vision, cognitive map, and directional decisions with limited time to navigate themselves in unfamiliar places [10]. Firefighters need to get complex spatial information within a short time for search and rescue [11]. Moreover, being more familiar with wayfinding training in dark environments can shorten wayfinding times [12], which increases the probability of mission success.

Data shows that the following seven factors endanger firefighters: building collapse, explosion (deflagration), flashover, fall or drop, electric shock, gas poisoning, and traffic accidents. Most firefighter deaths are due to explosion (37%) and building collapse (36%) [13]. Fire situations in confined spaces are particularly difficult [14]. Confined spaces in particular make it difficult to rescue victims because of the danger and complexity of such environments [15]. The physical characteristics of confined spaces, such as the size and shape of the environment, are strong determinants of mission difficulty. Confined space rescue is difficult because it places many psychological pressures upon rescuers, and so professional rescuers are essential to mission success [16]. Fires in underground areas involve various unique factors that problematize firefighting operations, such as greater inconvenience, more toxic smoke than usual, and difficulties in searching for and evacuating victims [17], while operations in environments such as high-rise buildings require an active air management strategy [18].

Firefighting entails a high workload and is a dangerous task, and the duration of a mission may be relative to self-contained breathing apparatus (SCBA) capacity. Additionally, firefighters can improve their ventilation efficiency and mission duration capacity by maintaining a high level of physical fitness [19]. The time required to respond to a fire mission is also a widely accepted performance indicator of mission success [20]. Given the hazards that firefighters face, protective gear is crucial for their survival. Escape situations are worsened by the decay of a firefighter’s thermal protective clothing (TPC) and SCBA [21,22]. As such, the less time a firefighter needs to navigate a fireground, the greater the chances are that the firefighter will be successful in doing so [23]. The findings of this study might provide strategies for improving the safety of firefighters working in dark and complex environments.

## 2. Methodology

### 2.1. Participants

Fifty-eight firefighters from the Fourth District of the NTFD participated in this study. All of them agreed to take part in the experiment and understood that the results would be released for publication. The Research Ethics Committee of the NTFD approved this study (R4-1070401, 13 February 2018).

### 2.2. Experimental Setting

**Figure 1:**
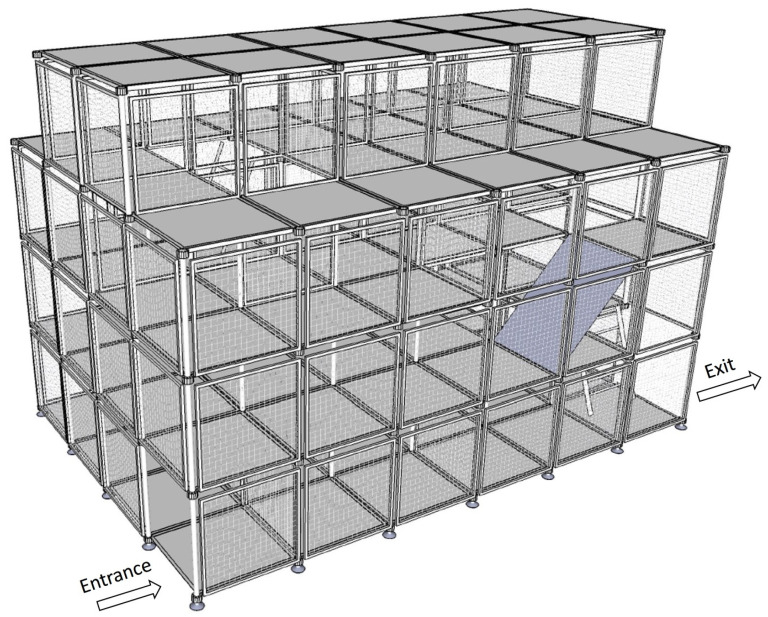
Perspective view of the DCEs.

The training facility utilized in this study was built by the NTFD. Figure 1 [24,25] shows a perspective view of the DCEs, the purpose of which is to train firefighters to face different obstacles in the DCEs, to simulate a fireground, and to test their ability to get out of trouble and escape confined spaces.

**Figure 2:**
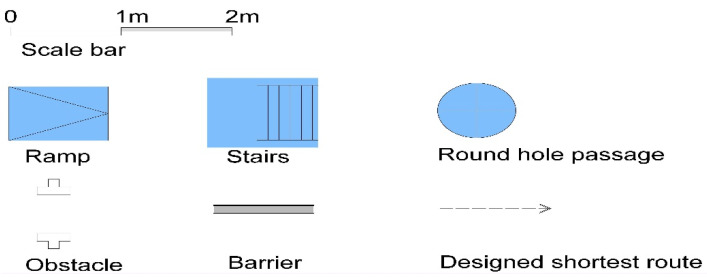
Legends for the floor plans.

**Figure 3:**
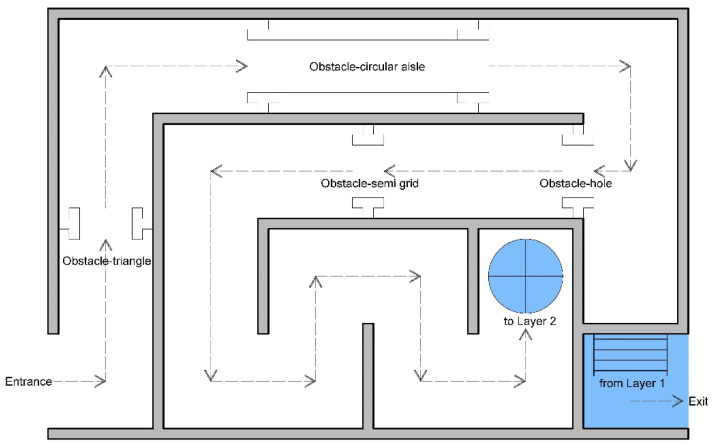
Floor plan for Layer 1.

**Figure 4:**
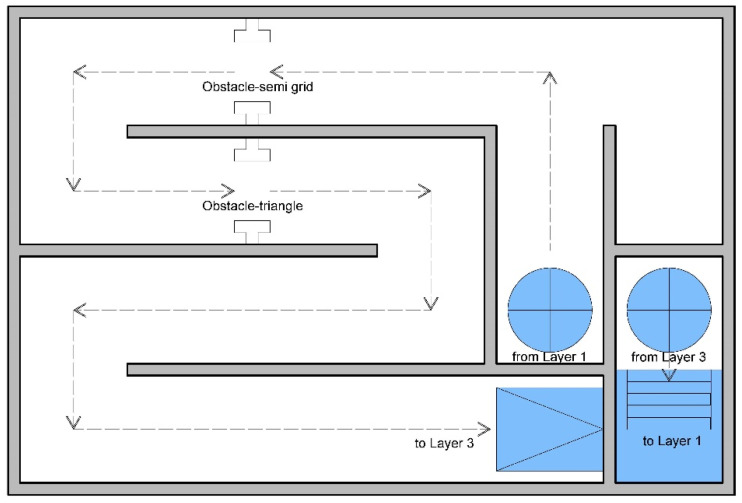
Floor plan for Layer 2.

**Figure 5:**
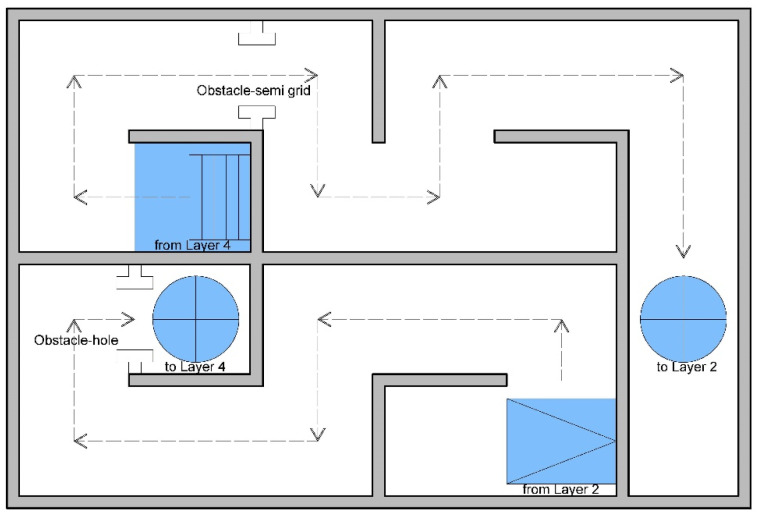
Floor plan for Layer 3.

**Figure 6:**
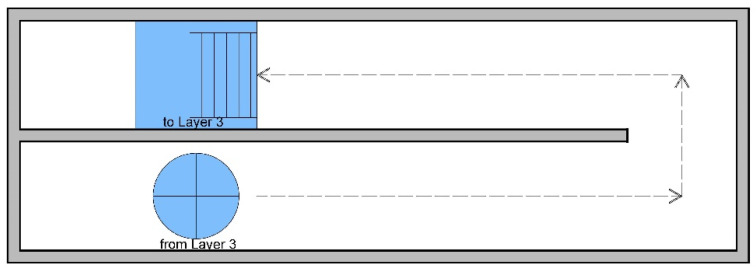
Floor plan for Layer 4.

Simulated fire scenarios in the DCEs include many obstacles on the wayfinding path. Figure 2, Figure 3, Figure 4, Figure 5 and Figure 6 are legends and a floor plan showing the numerous simulation paths and obstacles installed inside the DCE training facility, such as ramps, stairs, round hole passages, triangles, circular aisles, holes, squares, and semi-grid obstacles. The plan of the DCE comprises four layers, each of which is assembled with a confined space unit (CSU) of 100 ± 2 cm The CSUs in Layers 1–3 are approximately 400 × 600 × 300 cm, and Layer 4 is an additional 200 × 600 × 100 cm (W × L × H). Layers 1–3 each have 24 CSUs and Layer 4 has 12 CSUs, giving a total of 84 CSUs. The paths can be modified for different situations to suit different firefighter wayfinding training objectives.

### 2.3. Experimental Procedures

The experimental design was to simulate the DCEs that firefighters might encounter at an actual fireground. It was divided into three parts: (1) limited time for TPC and SCBA dressing, (2) limited time to perform four exercises, and (3) limited time for wayfinding in the DCEs.

For Part 1, the participants were required to wear full TPC and SCBA within 100 s, and then proceed to Part 2 immediately. The participants who could not finish part 1 within 100 s were not allowed to proceed to the next part.

**Figure 7:**
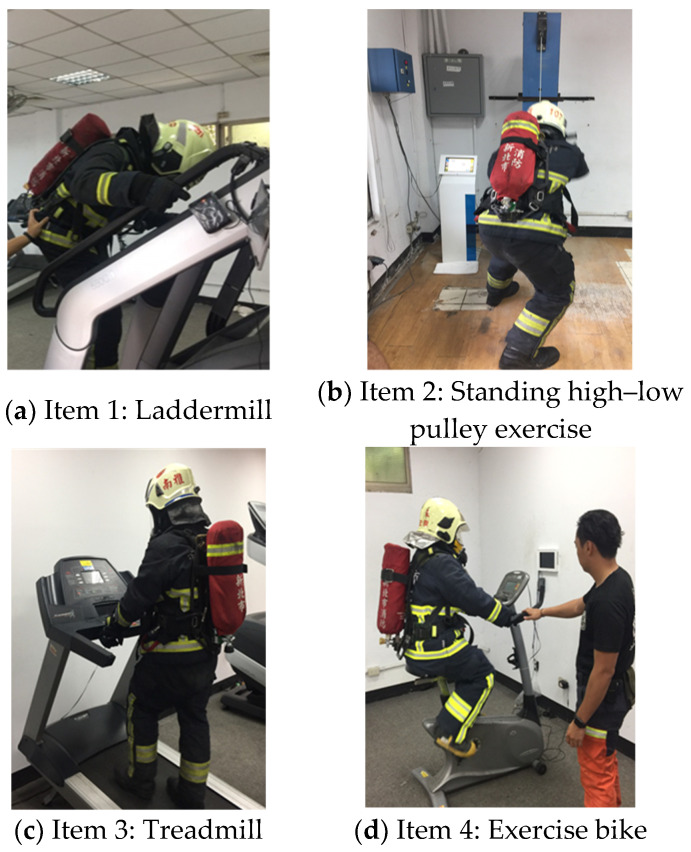
The four exercises for Part 2. (**a**) Laddermill (**b**) Standing high–low pulley exercise (**c**) Treadmill (**d**) Exercise bike.

For Part 2, the participants were required to complete four exercises within 7 min (including rest time). The participants who could not finish part 2 within the time limits or gave up in the process did not qualify for Part 3. The four exercises, which are shown in Figure 7, were:

- Item 1: Laddermill (rate adjusted to 19; approximately 20 m/min)
  Exercise time: 1 min
  Rest time: 30 s
- Item 2: Standing high–low pulley exercise (weight: 25 kg)
  Exercise time: 1 min
  Rest time: 30 s
- Item 3: Treadmill (slope = 15°, speed = 4 km/h)
  Exercise time: 1 min
  Rest time: 30 s
- Item 4: Exercise bike (speed fixed at 80 revolutions per minute)
  Exercise time: 2 min
  Rest time: 30 s

For Part 3, the participants’ wayfinding times were tracked from the moment they entered the DCEs until they reached the exit, at which point the times were recorded and the experiment was completed. Any participant who could not complete Part 3 within 15 min was considered injured or having aborted the mission, at which point the experiment was concluded.

### 2.4. Self-Report Survey

After finishing the experiment, the participants completed a self-report survey, which was discussed with the senior and experienced fire-training instructors to ensure the survey is practical and applicable for real firefighting situations at a fireground. After many meetings, the final version of the self-reported survey was formulated and applied in this study. The content of the survey was based on the following 13 factors: gender, age, fire station area (urban, rural, or mountain), years of service, rescue team training, handedness, starting orientation of wayfinding, experience of searching at firegrounds, fear of heights, fear of darkness, fear of the unknown, nervousness, and fear of confinement.

### 2.5. Statistical Methods

To check the results of the self-report survey and discuss which factor might be significant for the wayfinding time of firefighters in the DCEs, descriptive statistics were obtained with *t*-tests and multiple regressions were performed to check for differences in wayfinding times within the 13 factors. The multilinear regressions were conducted with stepwise model (S model), which removed the weakest correlated variable each time and left the factors that were suitable for the model. The *t*-tests and multiple regressions were conducted using IBM SPSS Statistics ver. 25.

## 3. Results

Overall, the mean wayfinding time of the 58 participants was 766.790 s (SD = 245.431). Table 1 shows a summary of the descriptive statistics. We performed *t* tests to check for differences in wayfinding times within 12 factors (gender was excluded). Comparisons between wayfinding times relative to the factors were performed between three groups within each factor. Bonferroni correction yielded an α level adjustment from 0.05 to 0.016. The *t* test results are given in the following:

(1) Gender: Only one female firefighter was in the sample of 58 participants. However, this reflects the low number of active female firefighters.

(2) Age: The age groups were ≤29, 30–34, and ≥35 years. The respective mean wayfinding times were 776.15, 689.58, and 866.05 s. Between the 30–34 and the ≥35 age groups, the difference was almost 3 min, while that between the 30–34 and ≤29 age groups was almost 1.5 min. The *t* test results were *t* (37) = 1.233, *p* = 0.225 for the ≤29 and 30–34 age groups; *t* (30) = −1.003, *p* = 0.324 for the ≤29 and ≥35 age groups; and *t* (43) = −2.317, *p* = 0.025 for the 30–34 and ≥35 age groups. The 30–34 age group had the shortest mean wayfinding time and was faster than the ≥35 age group, although this was only marginally significant, *t* (43) = −2.317, *p* = 0.025.

(3) Fire station area: Participants were divided according to where their fire station was located. The composition was 67.2% in urban areas, 32.8% in rural and mountain areas, *t* (56) = 0.268, *p* = 0.79. Firefighters working in rural fire stations had the shortest mean wayfinding time.

(4) Years of service: Participants were divided into three groups: 1–5 years (20.7%, Group 1), 6–10 years (53.4%, Group 2), and ≥11 (25.9%, Group 3) years of service. Participants with 1–5 years of service had the shortest mean wayfinding time (726.50 s). The *t* test results were *t* (41) = 0.629, *p* = 0.533 between Groups 1 and 2; *t* (44) = 0.333, *p* = 0.741 between Groups 2 and 3; and *t* (25) = 0.801, *p* = 0.431 between Groups 1 with 3.

(5) Rescue team training: Among the participants, 89.6% had received rescue team training. Those with rescue team training had a mean wayfinding time of 769.85 s, while those without training achieved a shorter mean wayfinding time of 740.33 s, *t* (56) = 0.277, *p* = 0.783.

(6) Handedness: Among the participants, 91.3% were right-handed and 8.6% were left-handed. Their respective mean wayfinding times were 759.60 and 843.00 s, *t* (56) = 0.277, *p* = 0.783.

(7) Starting orientation of wayfinding: Among the participants, 93.1% began their search along the right (mean wayfinding time = 773.31 s), 3.4% began their search along the left (562.50 s), and 3.4% began their search in a central direction (795.00 s). It seems that most people tended to start wayfinding along the right orientation. However, while these three groups did not show significant differences in wayfinding time, this may be due to the small sample.

(8) Experience searching at a fireground: Among the participants, 62.1% had previous experience of search and rescue under heavy smoke at a fireground. Their mean wayfinding time was 746.50 s, which was shorter than those without such experience, who had a mean of 800.00 s, *t* (56) = −0.803, *p* = 0.425.

(9) Fear of heights: Among the participants, 62.1% reported having a fear of heights. Their mean wayfinding time was 754.72 s, which was shorter than the mean of those with no fear of heights (786.55 s), *t* (56) = −0.476, *p* = 0.636.

(10) Fear of darkness: A total of 31.0% of the participants reported having a fear of dark environments. Their mean wayfinding time was 819.83 s, whereas those with no fear of dark environments had a mean of 742.93 s, *t* (56) = 1.106, *p* = 0.273.

(11) Fear of strangers: Among the participants, 32.8% reported being afraid of strangers. Their mean wayfinding time was 769.63 s, and those without this fear had a similar mean of 765.41 s, *t* (56) = 0.061, *p* = 0.952.

(12) Nervousness: Among the participants, 67.2% reported that they become nervous quickly. Their mean wayfinding time was 800.74 s, whereas those who reported that they do not get nervous easily had a mean wayfinding time of 697.11 s, *t* (56) = 1.527, *p* = 0.132.

(13) Fear of confinement: Firefighters with a fear of confinement (20.7%) had a mean wayfinding time of 902.25 s. Those without this fear had a mean wayfinding time of 731.46 s, revealing a marked difference of 170.79 s between the two groups, *t* (56) = 2.22, *p* = 0.031.

To further investigate the correlation among the factors, a multi-regression analysis was conducted. It aims to determine the weightings of different factors and determine whether multicollinearity exists among the factors. The “Stepwise-Model (S-model)” performs multiple regression many times. The weakest correlated variable is removed each time.

For the study, 12 factors were selected for multilinear regression to investigate which might affect the wayfinding performance of firefighters in the DCEs. Gender was excluded due to few female participants. After multiple regressions with the S model, the factor fear of confinement remained and was found to be significant (*p* = 0.031).

## 4. Discussion

The present study investigated 13 factors in 58 firefighters. The factors were gender, age, fire station area, years of service, rescue team training, handedness, starting orientation of wayfinding, experience of searching at firegrounds, fear of heights, fear of darkness, fear of the unknown, nervousness, and fear of confinement. The age factor showed a significant difference in wayfinding time. This indicates that fireground experience may be directly related to fire operation performance in the DCEs.

### 4.1. Personal Background and Fitness

Men accounted for 98.3% of the participants. The gender distribution of the research sample was in line with the NTFD population [26]. Another study pointed out that there is a need to target women’s strength and regulate support and facilities to reduce the risk of injury to female firefighters [27].

Age was a factor, but it related to a specific range. The younger age group of ≤29 years may not on average have had the same level of experience as those in the 30–34 age group, while those in the ≥35 age group may not on average have had the same level of peak fitness as their younger counterparts. Parts 2 and 3 of the experiment were designed to exhaust the participants in order to simulate their response to physical exertion during fireground operations and to then have them navigate through a dark environment. This was intended to test whether the participants possessed sufficient physical fitness for wayfinding in DCEs and the impact on their personal physical condition. A possible explanation for the results is that fitness levels may decrease with age, which can affect wayfinding performance in a DCE. Makrides et al. [28] investigated healthy people aged 15–70 years and found that when a person is in their 30 s, their total work declines linearly by about 6% per decade (R = −0.65).

TPC and SCBA, age, fatigue, and overtime duty can lead to loss of balance and injury. In particular, personal protective equipment (PPE) can make it difficult for some firefighters to stand upright. Sufficient experience and training can offset age-induced degradation of balance, which is associated with some injuries in firefighting operations. Implementing an objective assessment of the firefighters’ physical ability can help with establishing suitable training programs to help firefighters manage PPE properly [29,30,31].

### 4.2. Fire Station Area and Training

Different jurisdictions have different fire patterns and residential losses due to geographic and structural differences between urban and rural areas [32]. The characteristics of the location of a fire station differ between stations. These were categorized as rural, mountain, and urban areas. Statistically, due to the high incidence of fires in urban areas, the probability of a fire search and rescue event will be higher there than in other areas. However, the statistical test in the present study showed no significant difference in wayfinding times for this factor. In addition to improving physical fitness, rescue teams should also be trained to develop skills they will actually use in a rescue scenario, such as a rope rescue. However, searching a fireground is not the main objective of such training, and this may explain why the result for rescue team training was not significant.

### 4.3. Experience at Firegrounds

It seems reasonable to assume that years of experience can improve a firefighter’s decision-making and reactions to both anticipated and unanticipated situations at a fireground. To minimize the risk of injury or death, firefighters are required to implement standard operation guidelines on safety behaviors, to wear appropriate TPC and SCBA, and to avoid unsafe actions. The safety-oriented behaviors that develop with years of service can protect a firefighter. One previous study showed that effective communication, positive emotional interactions, and rapid adaptability could positively impact team effectiveness [33]. When considering a fire search task, firefighters from the same unit do not necessarily need to be organized into the same team for search and rescue tasks. Another study pointed out that when people become trapped at a fireground, quickly finding a relatively safe place and effectively exchanging messages with other firefighters could be a crucial challenge [34].

Safety behavior could be supported by fire departments having a positive safety climate [35,36]. Veteran firefighters have more experience and training than others. This supports the findings of a long-term study of firefighters that indicated that the longer a person is in the fire service, the more professional they will be when making critical decisions at a fireground [37].

Searching at a fireground is based on the procedure of one-way search. In a confined space with an exit, the exit will be found when the one-way search procedure is used, regardless of whether starting from the left or right. Therefore, with all other conditions considered equal, the time taken to navigate a fireground may vary according to the direction in which a person begins searching; thus, wayfinding may be affected by the design of the site. However, the DCEs in the present study were designed so that regardless of which side the participants approached the exits from, the outcome would not be affected by error or luck. Thus, the nonsignificant difference in the test results of handedness and starting orientation of wayfinding with wayfinding times are unsurprising.

Among the participants in the present study, 62% of them had experience searching at a fireground under heavy smoke. However, there was no significant difference in the test results between those who did and did not have such experience. The wayfinding completion times of those with more experience were not faster, probably because the fires faced by the firefighters were not completely dark or in confined spaces. Therefore, the experience of searching at a fireground under heavy smoke had no obvious benefit in the DCEs. In a previous study of escape simulation, the performance of fire safety professionals was not better than regular civilians, showing that previous experience might not necessarily be more helpful in certain emergency situations [38].

### 4.4. Mental Strength

The highest position of the DCEs is at least 3 m from the ground. Although 62% of the participants in the present study reported a fear of heights, there was no significant difference in their test results. This may be because the DCEs were dark and the participants were thus unable to observe a height difference. In addition, the participants took a low-post search approach and used their hands and feet to test for height differences in wayfinding to avoid the danger. People with a fear of heights will perceive heights to be a little higher than people without such a fear would. Overestimating the actual height when viewing from the top down is also usually associated with a fear of heights [39,40].

Most people, especially children, are afraid of the dark. Dark environments can cause potential dangers and risks that can lead to insecurities, tension, or anxiety [41]. Previous studies have suggested that human perception is affected by various lighting conditions, and a well-lit environment may increase a person’s sense of security. Ambient lighting has been recognized as a factor that might affect hazards and threats [42,43]. In the present study, 31% of participants reported being afraid of dark environments, but the test results showed no significant difference between those who did and did not have such fears. It may be that since the experiment required a large amount of physical energy consumption to complete, fear alone would not influence the results.

When considering a fire search task, firefighters from the same unit do not necessarily need to be organized into the same team search and rescue tasks. In the experiment, the teams of paired participants were randomly selected. Communication between teams searching a fireground is also a crucial element of success. Since the participants were paired with people who they were unfamiliar with, this study explored whether fear of the unknown would affect the wayfinding times. The test results revealed no significant difference, indicating that working with partners who the participants were unfamiliar with did not affect their communication while performing wayfinding.

Recent research showed that firefighting brings a modest burden of fear, but this does not appear to be related to wayfinding time [44]. In the present study, nervousness exhibited the second-highest explanatory power in the S model. Implementing various firefighting operations or searching at a fireground in high-temperature, high-pressure, or dark environments tests a firefighter’s ability to make correct decisions under pressure. Although a firefighter might be nervous in a dark environment, he or she generally would not be confused or overwhelmed to the same extent that regular civilians would. It is rigorous training on the rules of fire search and rescue that makes firefighters more able to complete this task. Firefighting is a profession with strict physical and mental requirements. Firefighters work under dangerous conditions, are exposed to extreme heat, are required to handle unpredictable situations, and are responsible for others’ lives and property. Hence, firefighting is a highly challenging job. Physiologically, wearing TPC and SCBA in an emergency makes physical effort more challenging and leads to a near maximum heart rate, high core temperature, and psychological stress [45,46,47,48,49,50,51,52].

Every year, workers are killed in incidents inside confined spaces [53]. Among the participants in the present study, 20.6% of them reported having a fear of confinement. Those with this fear had a mean wayfinding time of 902.250 s, whereas those without this fear had a mean wayfinding time of 731.457 s, showing a notable difference of 170.793 s. After multilinear regression with the S model, this was the only significant factor (*p* = 0.031). In confined spaces, people with claustrophobia are prone to a rapid heartbeat, trouble breathing, sweating, headache, nausea, and other symptoms. In worst-case scenarios, there would be feelings of panic. Because of the regular intensive training that firefighters go through, they are more able than civilians to overcome such symptoms, as well as psychological barriers in extreme environments.

### 4.5. Future Research

The limitations of this study might be that some factors were too small to consider meaningful in the statistical analysis. The gender of firefighters might be an interesting aspect for further research. Mental strength, which considers factors such as fear of darkness, fear of strangers, fear of confinement, fear of heights, and nervousness, is difficult to qualify. To enhance firefighters’ performance at firegrounds, more studies are needed to focus on firefighters’ safety in DCEs.

## 5. Conclusions

The results showed that age might be a marginally significant factor and fear of confinement might be a significant factor, which could affect the wayfinding time of firefighters in DCEs. Therefore, increasing firefighters’ wayfinding training in DCEs is recommended. Such training may compensate for a lack of relevant experience by allowing less-experienced firefighters to become more familiar with working in unfamiliar environments. Such training could not only improve the dark environment search experience, but also help firefighters avoid becoming lost in confined spaces at an actual fireground. Frequent training in DCEs should be conducted for all firefighters, especially those who have a fear of confined spaces.

## Figures and Tables

**Table 1:** Summary of descriptive statistics.

No.	Factor	Group	*n*	%	Mean	Min.	Max.	SD
Unit	-	-		-	(Seconds)	(Seconds)	(Seconds)	(Seconds)
1	Gender	Male	57	98.3%	767.28	295	1728	247.58
		Female	1	1.7%	739.00	-	-	-
2	Age	≤29 years	13	22.4%	776.15	505	1164	170.91
		30–34 years	26	44.8%	689.58	295	1225	221.85
		≥35 years	19	32.8%	866.05	510	1728	289.56
3	Fire station area	Urban	39	67.2%	772.87	455	1728	256.99
		Rural	8	13.8%	720.00	295	1225	273.35
		Mountain	11	19.0%	779.27	300	1019	194.94
4	Years of service	1–5 years	12	20.7%	726.50	541	990	124.64
		6–10 years	31	53.4%	791.42	470	1728	271.88
		≥11 years	15	25.9%	748.13	295	1225	267.47
5	Rescue team training	Yes	52	89.7%	769.85	295	1728	253.89
		No	6	10.3%	740.33	470	990	168.16
6	Handedness	Right-handed	53	91.4%	759.60	295	1728	247.26
		Left-handed	5	8.6%	843.00	615	1176	235.83
7	Starting orientation of wayfinding	Right	54	93.1%	773.31	295	1728	251.16
		Left	2	3.4%	562.50	541	584	30.41
		Central	2	3.4%	795.00	765	825	42.43
8	Experience of searching at a fireground	Yes	36	62.1%	746.50	295	1245	208.68
		No	22	37.9%	800.00	300	1728	298.43
9	Fear of heights	Yes	36	62.1%	754.72	295	1728	275.09
		No	22	37.9%	786.55	490	1225	191.61
10	Fear of darkness	Yes	18	31.0%	819.83	455	1728	271.84
		No	40	69.0%	742.93	295	1245	232.26
11	Fear of strangers	Yes	19	32.8%	769.63	295	1728	318.18
		No	39	67.2%	765.41	455	1245	205.90
12	Nervousness	Yes	39	67.2%	800.74	295	1728	253.04
		No	19	32.8%	697.11	300	1225	218.98
13	Fear of confinement	Yes	12	20.7%	902.25	455	1728	322.77
		No	46	79.3%	731.46	295	1245	211.34
